# Customized Pen for Patients With Arthritis

**DOI:** 10.7759/cureus.28797

**Published:** 2022-09-05

**Authors:** Rathod Prakash, Shreya Colvenkar, Ramesh Kunusoth, Aditya Mohan Alwala, Sampath Kumar

**Affiliations:** 1 Department of Oral and Maxillofacial Surgery, MNR Dental College & Hospital, Sangareddy, IND; 2 Department of Prosthodontics, MNR Dental College & Hospital, Sangareddy, IND

**Keywords:** pen holder, stiffness, pain, pen, arthritis

## Abstract

Patients with arthritis present with pain, stiffness and limited movement of fingers and hands. Performing tasks independently lead to a lot of anxiety and emotional trauma. Holding simple things like a toothbrush or pen is a cumbersome task. This article describes a simple technique to customize a pen for arthritis patients. The pen holder is customized to the patient's fingers making it easier to write.

## Introduction

Arthritis is a chronic disease [1] that commonly affects the hands and finger joints [2]. Simple tasks like holding a pen can be stressful because of pain, swelling and stiffness. Arthritis is associated with anxiety and depression [2-5], which further worsens the arthritis symptoms. The greatest degree of impairment is suffered by professional writers. It can be so emotionally disturbing for a professional writer that it deserves genuine attention. Customized pens would be a promising solution for such patients.

While designing a customized pen, all influencing factors, like the functional, emotional, social, and professional profile of the patient should be considered. Due to limited movement of hand and finger, it is very important to have a pen with an increased diameter that fits correctly in one’s hand to carry out writing correctly. A customized pen with increased volume provides a better grip on to the pen, making it easier to hold and write. This article describes a simple low-cost technique to customize a pen with materials readily available in a dental office.

## Technical report

Procedure

**Figure 1:**
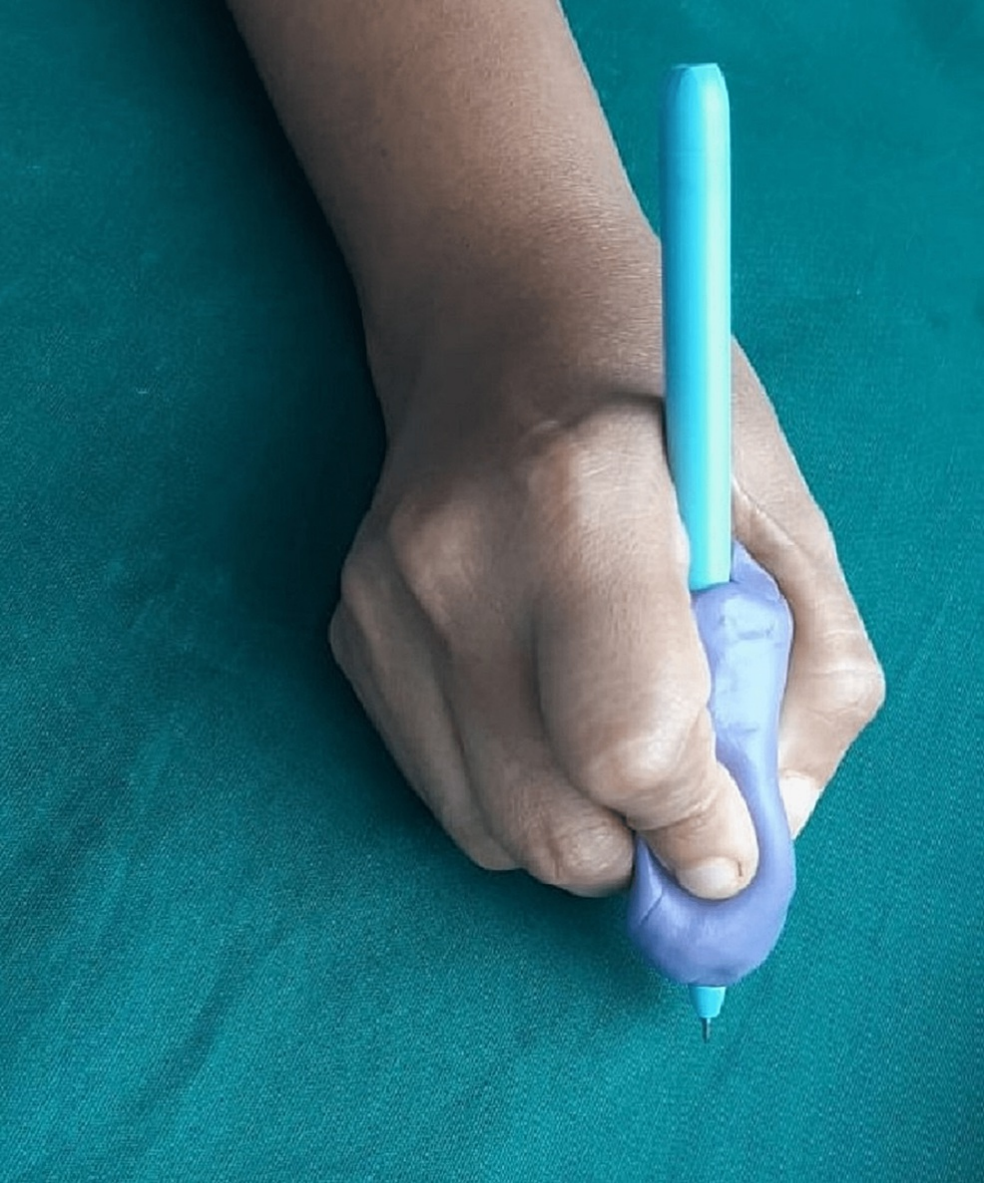
Molding of silicone putty impression material around the pen

Receive the patient with a caring attitude and explain in detail the procedure to be carried out. Grease the patient’s favorite pen with separating media (Vaseline, Hindustan Unilever Pvt. Ltd, India) to easily separate the pen from the impression material. Mold a dense silicone putty impression material (Photosil Soft Putty, Dental products of India, Mumbai, India) around the pen. Hold the pen in hand as if he/she is going to write (Figure 1).

**Figure 2:**
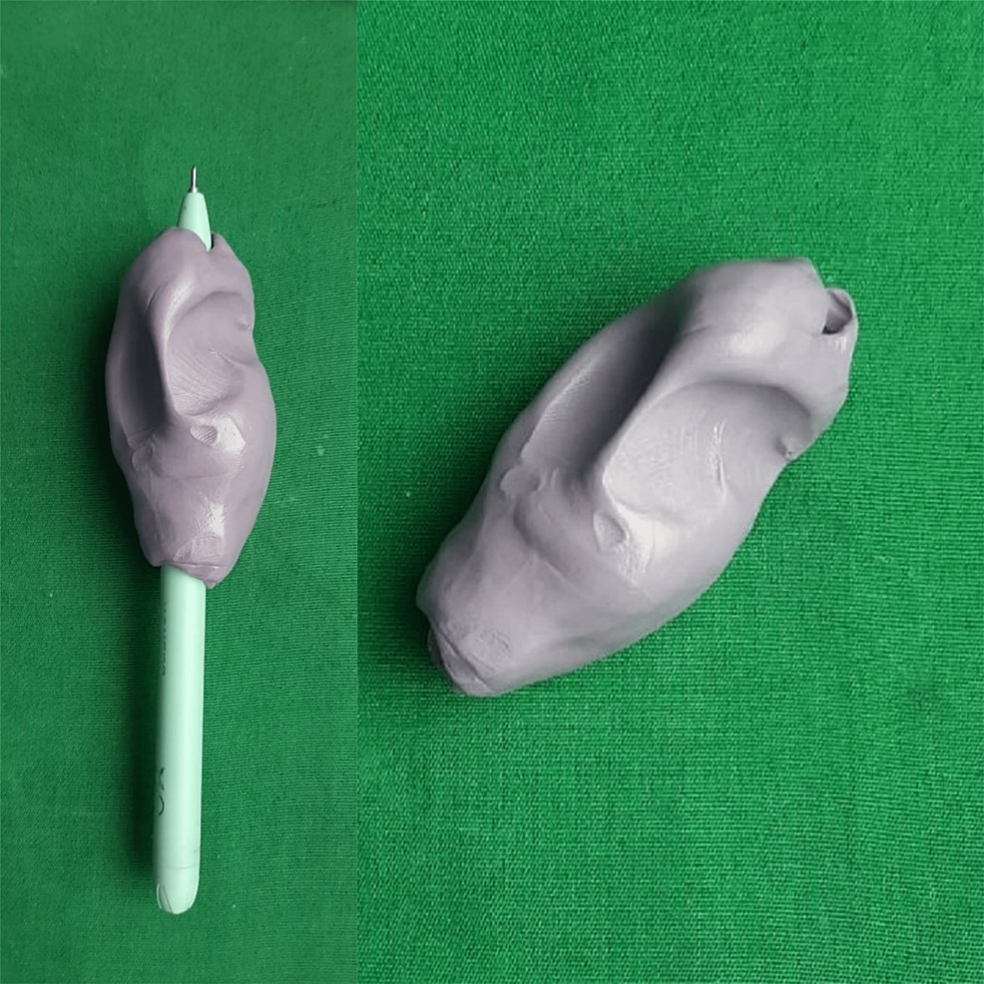
Molded silicone putty holder around the pen

Ask the patient to gently squeeze the material to get the shape of fingers in a position that is comfortable for writing. Remove the pen from the molded holder once the polymerization is complete (Figure 2).

**Figure 3:**
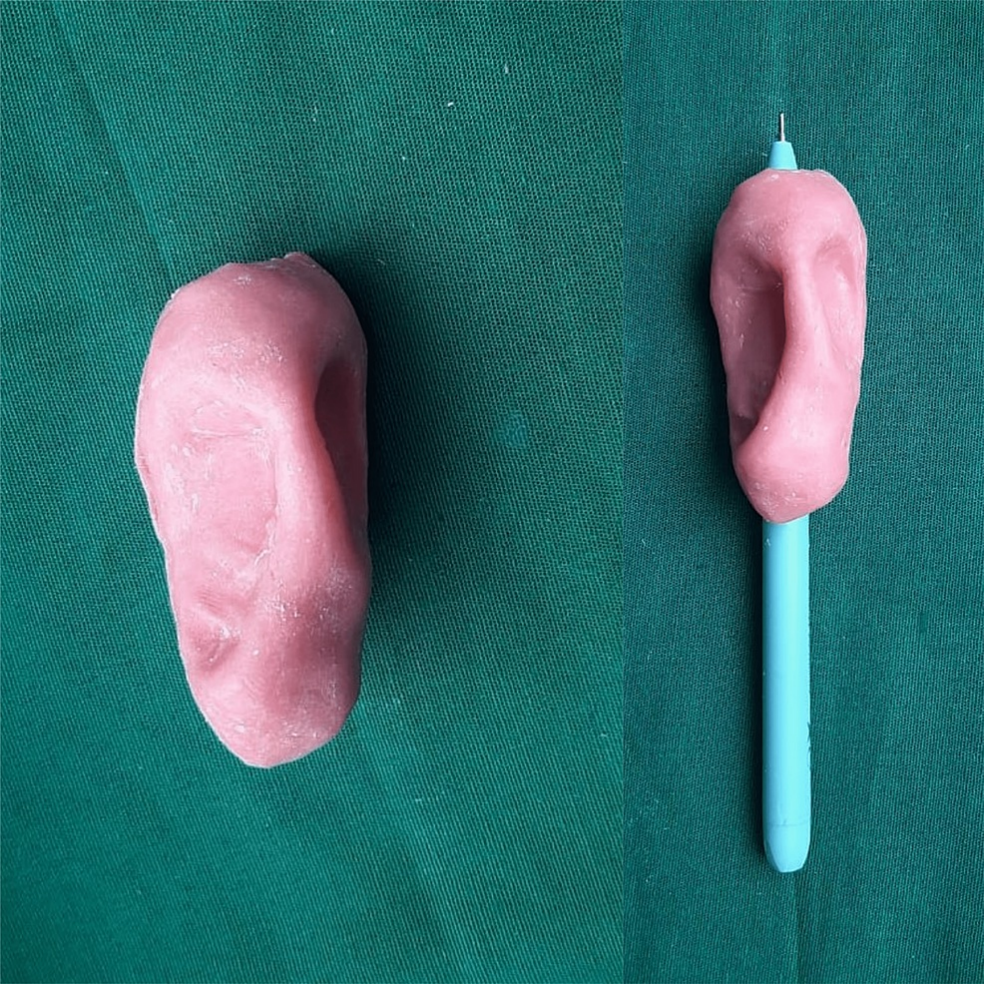
Acrylic holder for pen

Process the customized holder using heat polymerized acrylic resin (DPI Heat Cure, Dental Products of India Ltd.) with a conventional compression molding technique according to the manufacturer's instructions (Figure 3).

**Figure 4:**
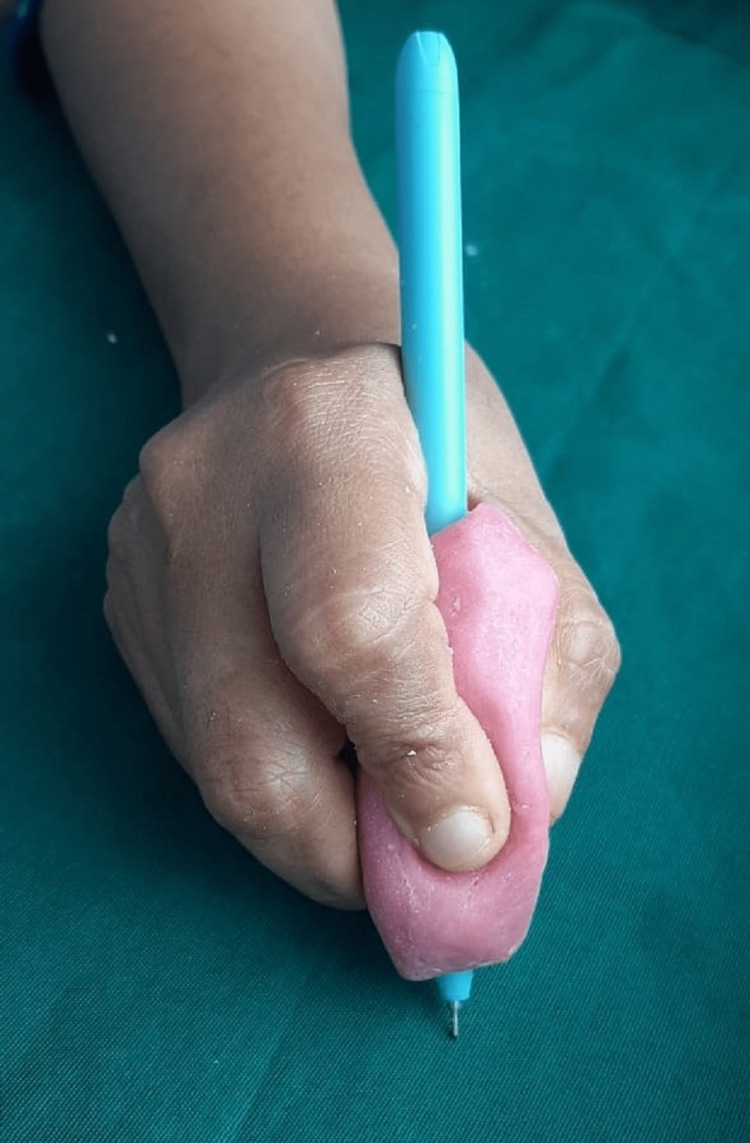
Customized acrylic pen holder

After finishing and polishing, insert the pen into the customized holder and ask the patient to write (Figure 4).

## Discussion

Arthritis patients suffer from joint pain, stiffness and limited mobility. Simple tasks like brushing teeth, writing, eating and even holding objects create significant emotional stress [2-4]. The best solution is to provide an alternative method to manage their disability and improve their quality of life. Various techniques to modify toothbrush handles [6-8] have been mentioned but the literature search does not mention any techniques for modification of pens.

The limited hand and finger movement associated with arthritis makes writing cumbersome and stressful. These patients need a pen that can make writing easier. The pen needs to be designed in such a way that it not only improves manual dexterity but also reduces writing fatigue. It should be designed from a material that is cheap and easy to grasp. Heat polymerized acrylic resin material was the best choice in this situation because of ease of manipulation and durability [6,8].

Several tools for pen holding like triangular grips, Bic XXL pen, Pilot Dr. Grip, Pen, Steady write pen, Arthwriter pen, and writing bird pen have been mentioned in the literature, but all these pens are not customized to individual hand and fingers [9]. This article describes a simple technique to customize the pen to individual fingers to improve the patient's writing skills. The customized pen holder was fabricated from heat polymerized acrylic resin which was easily available in the dental office. A wider diameter will make it easier to grasp by arthritis fingers thus minimizing the pressure while writing. This in turn will decrease the stress on the arthritis joint making handwriting legible. Further research needs to be carried out to understand the patient's experience and its impact on quality of life. The use of a customized pen can increase self-esteem and mental health by reducing reliance on caregivers.

## Conclusions

Many patients with arthritis or limited manual dexterity find difficulty in holding objects. A simple technique to customize a pen for patients with arthritis in the hands is presented. The customized holder with increased width will allow better grip, making writing easier.
